# Case Report: Successful Cerebral Revascularization and Cardiac Transplant in a 16-Year-Old Male With Syndromic *BRCC3*-Related Moyamoya Angiopathy

**DOI:** 10.3389/fneur.2021.655303

**Published:** 2021-03-30

**Authors:** Pierrick Pyra, Jean Darcourt, Marion Aubert-Mucca, Pierre Brandicourt, Olivier Patat, Emmanuel Cheuret, Karine Brochard, Annick Sevely, Lionel Calviere, Clément Karsenty

**Affiliations:** ^1^Pediatric Cardiology Unit, Department of Pediatrics, Children's Hospital, Toulouse University Hospital, Toulouse, France; ^2^Department of Diagnostic and Therapeutic Neuroradiology, Toulouse University Hospital, Hôpital Pierre Paul Riquet, Toulouse, France; ^3^Department of Medical Genetics, Toulouse University Hospital, Toulouse, France; ^4^Department of Neurosurgery, Toulouse University Hospital, Paul Sabatier University, Toulouse, France; ^5^Neurology Unit, Department of Pediatrics, Children's Hospital, Toulouse University Hospital, Toulouse, France; ^6^Nephrology Unit, Department of Pediatrics, Children's Hospital, Toulouse University Hospital, Toulouse, France; ^7^Department of Neurology, Toulouse University Hospital, Hôpital Pierre Paul Riquet, Toulouse, France; ^8^Toulouse Neuroimaging Center INSERM, UPS, Toulouse, France; ^9^Inserm U1048, Institut des Maladies Métaboliques et Cardiovasculaires (I2MC), Toulouse, France

**Keywords:** moyamoya angiopathy, revascularization, BRCC3, cardiac transplant, stroke

## Abstract

**Background:** *BRCC3/MTCP1* deletions are associated with a rare familial moyamoya angiopathy with extracranial manifestations.

**Case:** We report the case of an adolescent male presenting with progressive and symptomatic moyamoya angiopathy and severe dilated cardiomyopathy caused by a hemizygous deletion of *BRCC3/MTCP1*. He was treated for renovascular hypertension by left kidney homograft and right nephrectomy in infancy and had other syndromic features, including cryptorchidism, growth hormone deficiency, and facial dysmorphism. Due to worsening of the neurological and cardiac condition, he was treated by a direct superficial temporal artery to middle cerebral artery bypass to enable successful cardiac transplant without cerebral damage.

**Conclusions:** *BRCC3*-related moyamoya is a devastating disease with severe heart and brain complications. This case shows that aggressive management with cerebral revascularization to allow cardiac transplant is feasible and efficient despite end-stage heart failure.

## Introduction

Moyamoya is a rare cerebrovascular angiopathy characterized by progressive stenosis of the terminal part of the intracranial carotid arteries and/or proximal middle or anterior cerebral arteries. These lesions lead to the development of abnormal and fragile collateral vessels and are responsible for ischemic and hemorrhagic stroke (1). Moyamoya angiopathy can be associated with various conditions, including neurofibromatosis, Down syndrome, radiotherapy, sickle cell disease, and rare *BRCC3/MTCP1* deletions that associate a rare X-linked moyamoya syndrome with multisystemic manifestations (MIM #300845) (2).

## Case Report

### Background

The male patient was born prematurely at 34 weeks gestation at a weight of 1,700 g, with a history of intrauterine growth retardation against the background of bridged left renal fibrodysplasia in the mother. Cryptorchidism and mild pulmonary valve stenosis were diagnosed at birth.

He had an older brother, born at 32 weeks gestation, who had spastic diplegia, moderate intellectual disability, perception deafness, and growth hormone deficiency without myocardiopathy. There was no familial history of moyamoya disease.

### History

The patient had growth retardation at −2.7DS, with partial growth hormone (GH) deficiency but no hypogonadism (delayed puberty). During infancy, hypertension was discovered during his heart monitoring. Further investigations revealed bilateral stenosis of the renal arteries with a small right kidney. The aortic angio-magnetic resonance imaging (MRI) found evidence of midaortic syndrome with diffuse dysplasia, and stenosis of the inferior renal aorta and bilateral renal arteries. Williams-Beuren syndrome was ruled out.

The hypertension quickly became resistant despite a four-drug regimen. He underwent multiple (right and/or left) angioplasties in childhood without improvement or with rapid restenosis, unsuccessful stent in the left renal artery, then a right nephrectomy for atrophic right kidney. Finally, during adolescence, he underwent a major surgery with insertion of an aorto-aortic Dacron tube and left kidney homograft (on the left common iliac artery). During this surgery, he experienced low systemic flow, which induced left hemiparesis. The cerebral MRI showed severe hypoperfusion of the bilateral corona radiata, with watershed ischemic lesions associated with dysplasia of both internal carotid terminations, suggesting moyamoya angiopathy. The patient recovered well. No surgical revascularization was planned because of the atypical (because of diffuse stenosis and few collaterals) moyamoya syndrome, the absence of recurrent stroke, the normal perfusion sequences, the absence of MRI progression during follow-up and recurrence of neurological symptoms. Reanalysis of *PTEN, SOS1, RAF1*, and *SHOC2* based on the hypothesis of Noonan syndrome, and analysis of *ACTA2*, given the association of moyamoya angiopathy and the clinical presentation, did not identify any pathogenic variants.

The hypertrophic hypertensive cardiomyopathy progressed to mild dilated cardiomyopathy with a left ventricular ejection fraction (LVEF) of 50%.

Three years later, the patient presented with a transient ischemic attack (TIA) without significant modification on MRI (non-contributive perfusion sequences, stable stenosis of M1) and normal blood pressure. He was hospitalized 3 weeks later for faintness associated with cardiac decompensation, hypotension, and worsening of the echocardiographic parameters (LVEF 20%, post-capillary systolic pulmonary hypertension at 55 mmHg, moderate mitral regurgitation). He was no more hypertensive thereafter. The association of moyamoya disease, dilated myocardiopathy, renovascular hypertension, GH deficiency, cryptorchidism, and mild dysmorphism prompted investigation for the *BRCC3/MTCP1* deletion.

**Figure 1:**
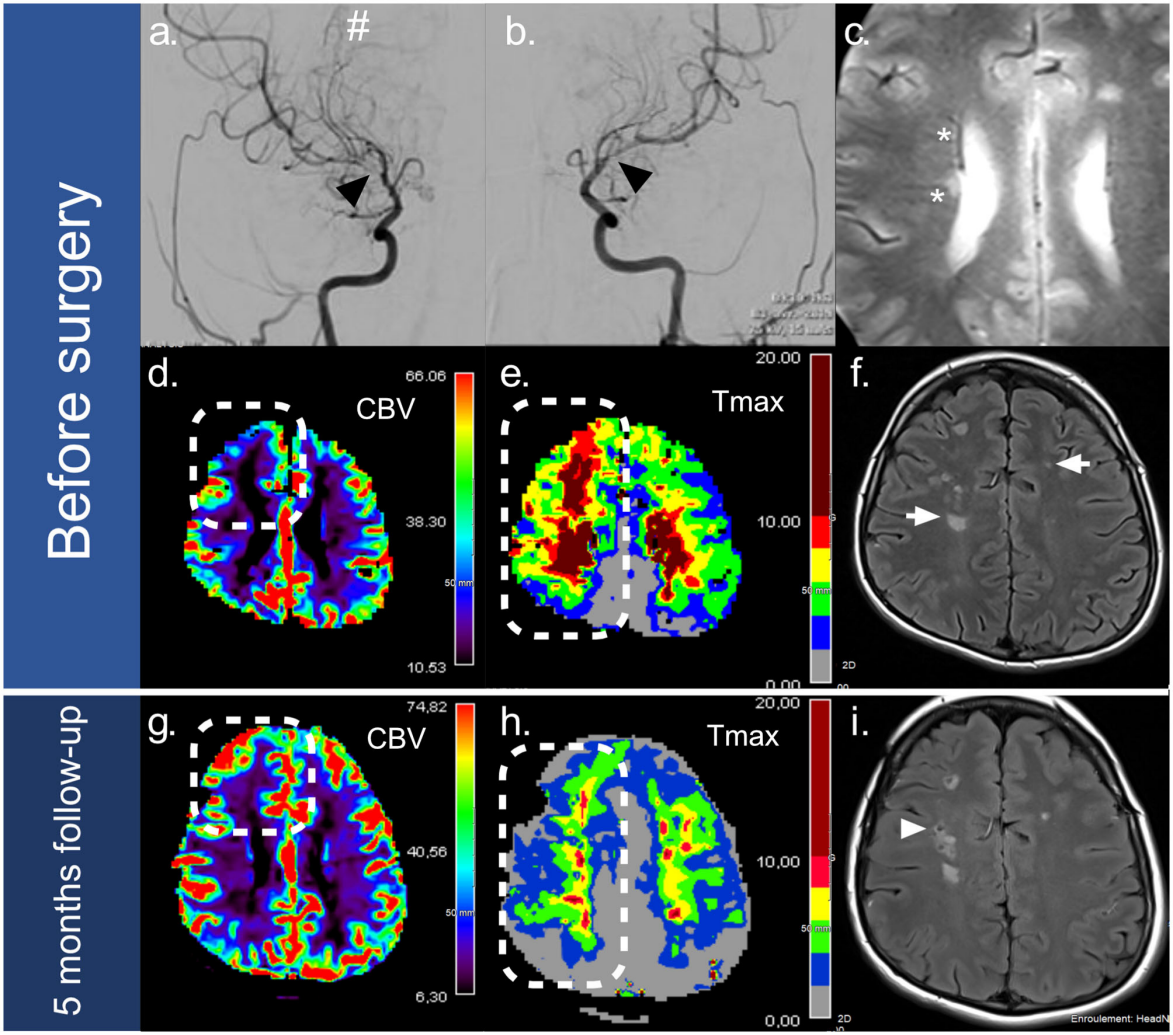
Imaging of moyamoya lesions and 5-month follow-up. Before surgery, moyamoya lesions were observed on the right **(a)** and left **(b)** internal carotid artery angiograms, revealing bilateral stenosis of the internal carotid arteries termination (black arrows), with moyamoya collaterals (#). Magnetic resonance imaging **(c–f)** found asymmetric enlargement of the medullary veins in relation with cerebral hypoperfusion (*) visible on the T2-GE sequence **(c)**, associated with bilateral watershed ischemic lesions on FLAIR **(f)** (white arrow). Perfusion imaging pointed out severe hypoperfusion in the right frontal junctional territory (squared dots) on the cerebral blood flow cartography (CBV) **(d)** and the TMAX cartography **(e)**. A five-month follow-up scan **(g–i)** showed significant improvement of the left frontal lobe perfusion after surgery with: (1) an increase in cerebral blood flow (**d** vs. **g**) (squared dots), and (2) shrinkage of delayed perfusion areas on TMAX >6s (**e** vs. **h**) (rectangular dots). No recurrent ischemic lesions were observed after surgery. Preoperative ischemic lesions progressed into lacunar lesions (**f** vs. **i**) (white arrowhead).

Two months later, he developed several episodes of transient left hemiplegia with ischemic lesions in the internal carotid artery area. A cerebral angiogram showed worsening of the M1 stenosis and occlusion of the two anterior cerebral arteries (Figures 1a,b). Perfusion MRI showed severe bilateral frontal hypoperfusion of cerebral blood flow, and Tmax cartography (Figures 1d,e). Neurologic recovery was excellent except for persistent mild ataxia and melokinetic apraxia.

We discussed at this time the feasibility of revascularization and need of cardiac transplant because he remained in end-stage heart failure despite optimal cardiac therapy. Arterial tension objective was minimum 126/77 mmHg. The feasibility and the risk of surgical cerebral revascularization and the risk of a large stroke during cardiac transplant [because of the cerebral hemodynamic status, embolic complications, arrest, tamponade, and the need for postoperative extracorporeal life support (ECLS)] were weighed. Therefore, in order to improve cerebral perfusion to allow for a cardiac transplantation, we decided to perform direct cerebral revascularization surgery.

A superficial temporal artery to middle cerebral artery (STA-MCA) bypass was performed on the right hemisphere related to the clinical symptoms. We used indocyanine green fluorescence angiography during anastomosis to evaluate the bypass blood flow. Surgery was preceded by an inotropic infusion (levosimendan).

No peri- or postoperative complications occurred. He did not experience further recurrent TIAs. Five months of follow-up imaging with conventional and perfusion-weighted imaging showed that the anastomosis was efficient, with a decrease in frontal hypoperfusion and no additional ischemic lesion. Figure 1 illustrates the increase in brain perfusion on cerebral blood flow cartography and the shrinkage of delayed perfusion areas (TMAX >6s) on TMAX cartography, which was enabled by the postoperative development of collateral vessels (Figures 1g,h).

**Figure 2:**
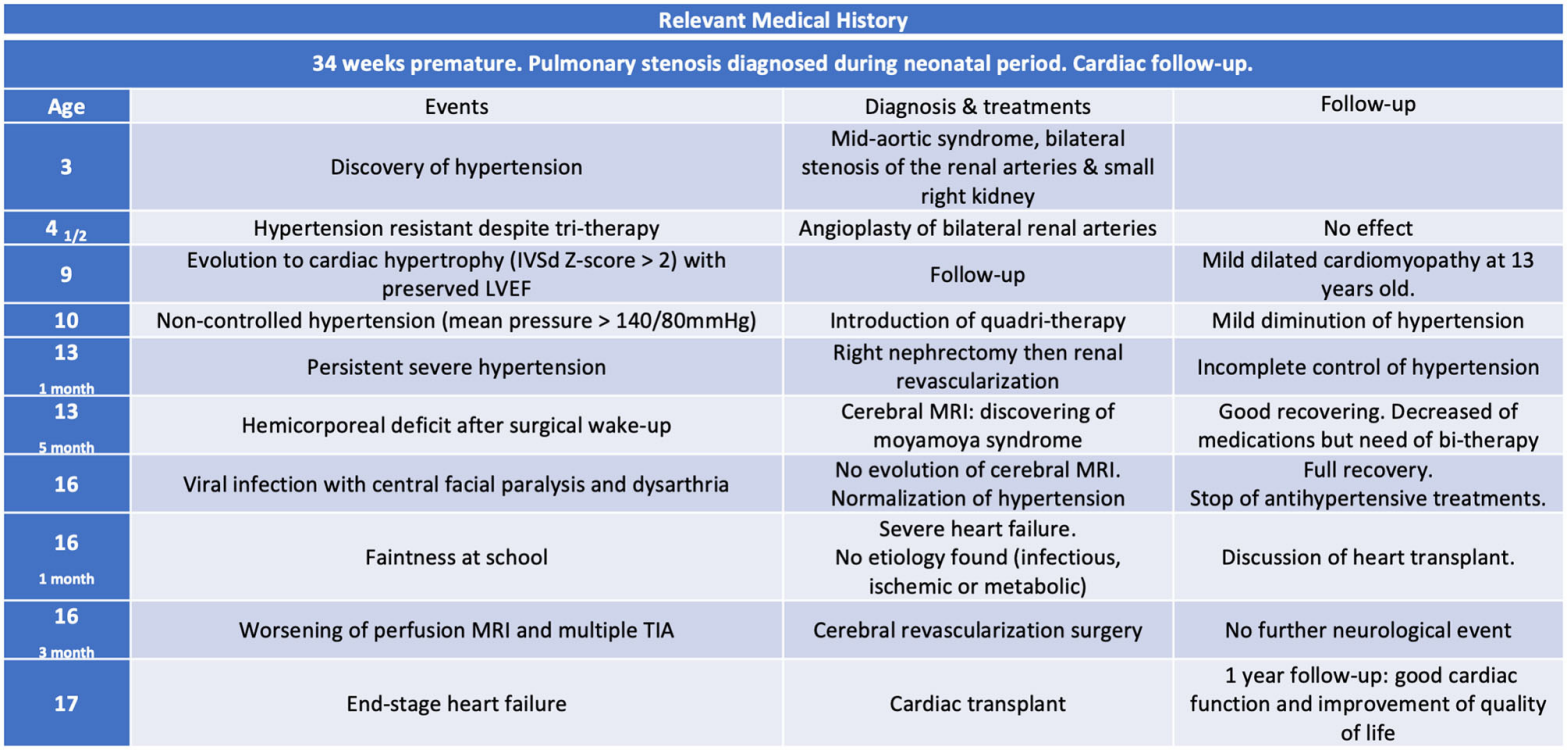
Timeline table, resuming evolution of neurological and cardiac disease. The patient's written consent was obtained for publication.

The patient was registered on the cardiac transplant list 2 months later. New episodes of cardiac decompensation after the neurosurgical intervention required treatment with levosimendan. He underwent a heart transplant a few days before reaching adulthood, at a weight of 30 kg. The last follow-up, 1 year after the transplant, was excellent (Figure 2). He began to ride a bike and walk hundreds of meters, he gained weight and muscle, and the last echocardiography showed normal cardiac function (LVEF 62%).

## Discussion

Nine *BRCC3*-related moyamoya patients from three families (2–4) have been reported in the literature with a syndromic presentation including growth retardation (9/9), moyamoya angiopathy (8/9), hypergonadotropic hypogonadism (7/9), partial GH deficiency (4/5), early-onset cataracts (4/5), dilated myocardiopathy (3/7), renovascular hypertension (3/5), coronaropathy (1/9), and dysmorphism dominated by hypertelorism syndrome with a syndromic presentation including craniofacial dysmorphism and premature graying of hair. The age of onset of the neurological symptoms was variable, from 4 to 32 years. This is the first case, to our knowledge, to have presented diffuse dysplasia and stenosis of the inferior renal aorta and pulmonary valve stenosis. Very little information has been published on the management of these patients.

*BRCC3* plays an important role in angiogenesis, and moyamoya vasculopathy with mutation of *BRCC3* is a diagnosis to be kept in mind in the event that prior analysis with a compatible phenotype is negative.

Symptomatic moyamoya angiopathy is often managed with surgical revascularization to improve cerebral perfusion (direct technique with surgical anastomosis or indirect technique with synangiosis). Heart failure is associated with increased mortality in non-cardiac surgery (5, 6). Anesthesia involves many changes in physiology, and the postoperative state is a vulnerable period similar to a cardiac stress test. Prior preparation and complete monitoring during the neurosurgical procedure are also essential, as is the careful choice of anesthetic drugs. The risk of periprocedural stroke is high, notably because of hypotension during the induction of general anesthesia. Nevertheless, we thought that it was the only choice for preventing recurrent hemodynamic stroke and for protecting the brain from ischemia during hemodynamic stress related to cardiac transplant.

On the other hand, cardiac transplantation is also associated with a high risk of stroke and functional decline during the perioperative period (7–9).

This case shows that in the context of this devastating disease, multidisciplinary, and aggressive management with cerebral revascularization followed by cardiac transplant is feasible and efficient despite end-stage heart failure.

## Data Availability Statement

The raw data supporting the conclusions of this article will be made available by the authors, without undue reservation.

## Ethics Statement

Ethical review and approval was not required for the study on human participants in accordance with the local legislation and institutional requirements. Written informed consent to participate in this study was provided by the participants' legal guardian/next of kin.

## Author Contributions

PP wrote the case report. JD made the figure and performed the endovascular part. MA-M and OP performed the genetic part. PB the neurosurgical part. EC and KB were involved in the pediatric management and pediatric supervision. AS helped for the neuroimaging. LC made the supervision. CK rewrite the manuscript and validate each part. All authors contributed to the article and approved the submitted version.

## Conflict of Interest

The authors declare that the research was conducted in the absence of any commercial or financial relationships that could be construed as a potential conflict of interest.
